# Clinical and biochemical phenotypes of Lean MAFLD: machine learning-based biomarker identification and statistical association analysis in a cross-sectional study

**DOI:** 10.3389/fpubh.2026.1835144

**Published:** 2026-07-31

**Authors:** Hui Zhang, Yansong Ji, Guilian Chen, Zhen Tian

**Affiliations:** 1 Department of Neurology, Huai'an Hospital Affiliated to Yangzhou University, Huai'an, China; 2 Health Management Center, The Affiliated Huai'an No. 1 People's Hospital of Nanjing Medical University, Huai'an, China

**Keywords:** biomarkers, clinical phenotype, cross-sectional study, Lean MAFLD, machine learning, metabolic dysfunction-associated fatty liver disease, public health

## Abstract

**Background:**

Metabolic dysfunction-associated fatty liver disease (MAFLD) presents a significant public health issue in China. The “lean” subtype (L-MAFLD) accounts for a certain proportion of cases but is frequently underrecognized in primary care due to the absence of visible obesity. Its distinct clinical and biochemical phenotype remains unclear. This study aimed to identify routine biomarker features and statistical associations related to the metabolic variability of L-MAFLD within the general population.

**Methods:**

This retrospective, cross-sectional study was conducted from January 2023 to January 2025 and included 12,553 adults who underwent health checkups at a tertiary hospital in Jiangsu, China. Participants were divided into groups based on body mass index (BMI) and ultrasonography: L-MAFLD (*n* = 330), overweight/obese MAFLD (*n* = 9,207), and non-MAFLD controls (*n* = 3,016). An integrated machine learning framework—including least absolute shrinkage and selection operator, extreme gradient boosting, and Boruta—was used to identify candidate physiological and biochemical variables. These biomarkers were then statistically evaluated using multivariable Firth-penalized logistic regression. Sensitivity analyses included age matching and additional adjustment for the available metabolic syndrome (MetS) component count. To assess non-linear associations and potential effect modification, restricted cubic splines (RCS) and stratified analyses were performed.

**Results:**

The machine learning pipeline initially identified 26 variables. After evaluating stability and redundancy, 19 variables remained in the final models. Higher levels of pulse rate, fasting blood glucose (FBG), alkaline phosphatase, and prealbumin were independently associated with higher odds of L-MAFLD. Higher levels of the alanine aminotransferase to aspartate aminotransferase (ALT/AST) ratio, serum creatinine (Scr), free triiodothyronine (FT3), diastolic blood pressure, and white blood cell count were associated with lower odds of L-MAFLD. Sensitivity analyses generally supported the main findings, although FT3 lost significance after age matching, and FBG was attenuated after adjustment for the MetS component count. RCS analysis showed an L-shaped nonlinear relationship between Scr and L-MAFLD, with a key inflection point at 60 μmol/L. A significant sex-specific interaction was found for the ALT/AST ratio (*P* = 0.012).

**Conclusion:**

L-MAFLD shows a distinct clinical and biochemical phenotype, indicating metabolic imbalance despite normal BMI. These routine biomarkers may serve as potential epidemiological indicators of metabolic vulnerability among normal-weight individuals. The findings remain association-based. Future longitudinal multicenter studies with more complete metabolic profiling and external validation are needed.

## 1 Introduction

Metabolic dysfunction-associated fatty liver disease (MAFLD) is a diagnostic concept that highlights the link between hepatic steatosis and metabolic dysfunction, including insulin resistance (IR) and abnormal carbohydrate and lipid metabolism (1). MAFLD has now surpassed chronic viral hepatitis to become China's leading chronic liver disease and is the primary cause of elevated liver enzymes among health checkup populations (2). The lean subtype of MAFLD (L-MAFLD) represents a specific phenotype within this spectrum. An international consensus defines L-MAFLD as MAFLD occurring in individuals with a body mass index (BMI) below 23 kg/m^2^ in Asian populations, accounting for approximately 5–30% of all cases (3, 4). Obesity remains the primary risk factor for MAFLD. Most research has focused on overweight or obese individuals with MAFLD (O-MAFLD).

Individuals with L-MAFLD have normal weight or a thin physique and lack the traditional obesity phenotype. They may be overlooked in primary care. Multiple studies indicate that these individuals may experience faster liver fibrosis progression and face a higher risk of cardiovascular, renal, and metabolic complications than those with O-MAFLD (5–7). This higher-risk profile deserves greater attention in public health and primary care. The routine biomarker features of L-MAFLD, particularly relative to O-MAFLD, remain unclear. Characterizing routine, easily accessible biomarkers may help clarify this clinical phenotype and generate hypotheses for future validation. This study aimed to identify routine biomarker features and characterize statistical associations related to the metabolic variability of L-MAFLD in the general population.

## 2 Materials and methods

### 2.1 Participants

**Figure 1:**
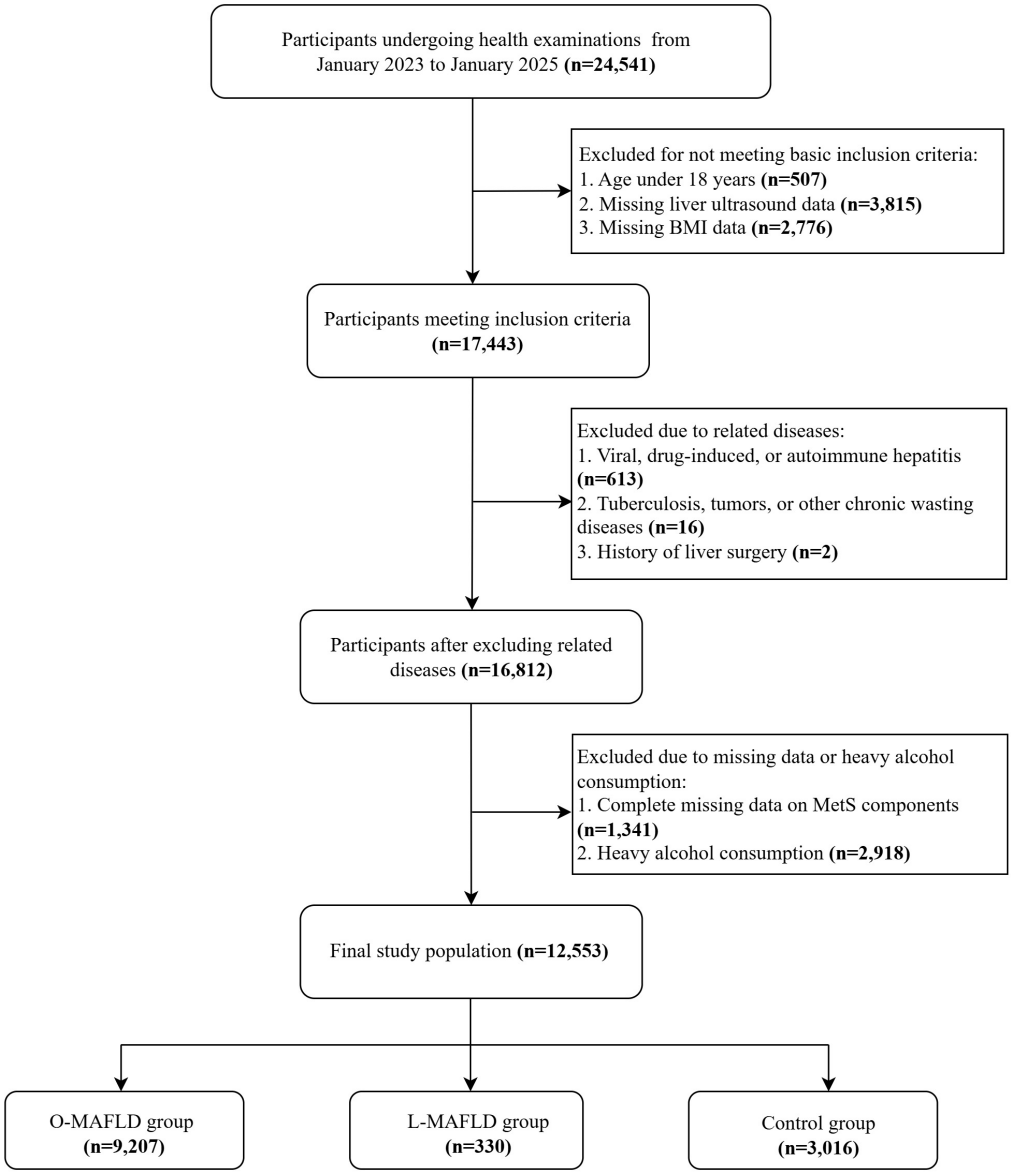
Flowchart of participant selection. BMI, body mass index; L-MAFLD, lean metabolic dysfunction-associated fatty liver disease; MetS, metabolic syndrome; O-MAFLD, overweight/obese metabolic dysfunction-associated fatty liver disease.

This cross-sectional study involved adults undergoing routine health checkups at the Health Management Center of the Affiliated Huai'an No. 1 People's Hospital of Nanjing Medical University from January 2023 to January 2025. Although the center handles a large annual volume of routine health checkups, this study focused on an initial pool of 24,541 individuals who specifically selected a comprehensive metabolic assessment package, comprising a fasting biochemical panel and abdominal ultrasonography. This study was reported in accordance with the STROBE guidelines. Data were retrospectively extracted from the electronic health record system to identify eligible participants. Inclusion criteria were: (1) aged ≥ 18 years; (2) having undergone hepatic ultrasonography; and (3) complete BMI data. Exclusion criteria included: (1) diagnosis of viral hepatitis, drug-induced hepatitis, autoimmune hepatitis, or other specific liver diseases; (2) presence of tuberculosis, tumors, or other chronic wasting diseases; (3) history of liver surgery; (4) missing data on all components of metabolic syndrome (MetS); and (5) heavy alcohol intake (≥ 210 g/week for men, ≥ 140 g/week for women). A total of 12,553 eligible participants were included in the final analysis (Figure 1). The study was approved by the Medical Ethics Committee of the Affiliated Huai'an No. 1 People's Hospital of Nanjing Medical University (Approval ID: KY-2025-123-01), and the requirement for written informed consent was waived by the ethics committee due to the retrospective nature of the study.

### 2.2 Clinical parameters

The following candidate clinical physiological indicators and biomarkers were included for analysis: (1) Demographic covariates: sex, age; (2) Basic cardiovascular physiological indicators: systolic blood pressure (SBP), diastolic blood pressure (DBP), pulse; (3) Lipid metabolism markers: total cholesterol (TC), lipoprotein(a) [Lp(a)], triglycerides (TG), high-density lipoprotein cholesterol (HDL-C), low-density lipoprotein cholesterol (LDL-C); (4) Glucose metabolism markers: fasting blood glucose (FBG), hemoglobin A1c (HbA1c); (5) Liver function markers and enzymes: alanine aminotransferase (ALT), aspartate aminotransferase (AST), the alanine aminotransferase to aspartate aminotransferase ratio (ALT/AST), γ-glutamyl transferase (GGT), lactate dehydrogenase (LDH), α-hydroxybutyrate dehydrogenase (HBDH), albumin (ALB), globulin (GLB), total bilirubin (TBil), direct bilirubin (DBil), indirect bilirubin (IBil), and serum prealbumin (PAB); (6) Serum electrolytes and minerals: potassium, sodium, chloride, calcium, phosphorus; (7) Kidney function indicators: uric acid (UA), blood urea nitrogen (BUN), serum creatinine (Scr), cystatin C (CysC); (8) Additional biochemical markers: alkaline phosphatase (ALP), creatine kinase (CK); (9) Complete blood count parameters: hemoglobin (Hb), red blood cell count (RBC), white blood cell count (WBC); (10) Thyroid markers: free triiodothyronine (FT3), free thyroxine (FT4), thyroid-stimulating hormone (TSH).

### 2.3 Diagnostic criteria and grouping definitions

According to current consensus and guidelines, MAFLD was diagnosed based on ultrasonographic evidence of hepatic steatosis in combination with at least one metabolic abnormality. Hepatic ultrasonography was performed by experienced sonographers who were blinded to participants' clinical and laboratory data, using standard diagnostic criteria for hepatic steatosis. Metabolic abnormalities included overweight or obesity (BMI ≥ 23 kg/m^2^), type 2 diabetes mellitus, or at least two components of MetS. The specific MetS criteria were defined as follows: (1) waist circumference ≥ 90 cm in men or ≥ 80 cm in women; (2) SBP ≥ 130 mmHg or DBP ≥ 85 mmHg; (3) HDL-C < 1.0 mmol/L in men or < 1.3 mmol/L in women; (4) TG ≥ 1.7 mmol/L; and (5) FBG ≥ 5.6 mmol/L or HbA1c ≥ 5.7%. Participants were divided into three groups based on BMI and MAFLD criteria: the L-MAFLD group (*n* = 330), comprising individuals with BMI < 23 kg/m^2^ who had ultrasonographic hepatic steatosis together with type 2 diabetes or at least two MetS components; the O-MAFLD group (*n* = 9,207), comprising individuals with BMI ≥ 23 kg/m^2^ and ultrasonographic hepatic steatosis; and the non-MAFLD control group (*n* = 3,016), comprising individuals with BMI < 23 kg/m^2^ without MAFLD. Ultrasonographic confirmation of hepatic steatosis was required for inclusion in both the L-MAFLD and O-MAFLD groups.

### 2.4 Procedure

**Figure 2:**
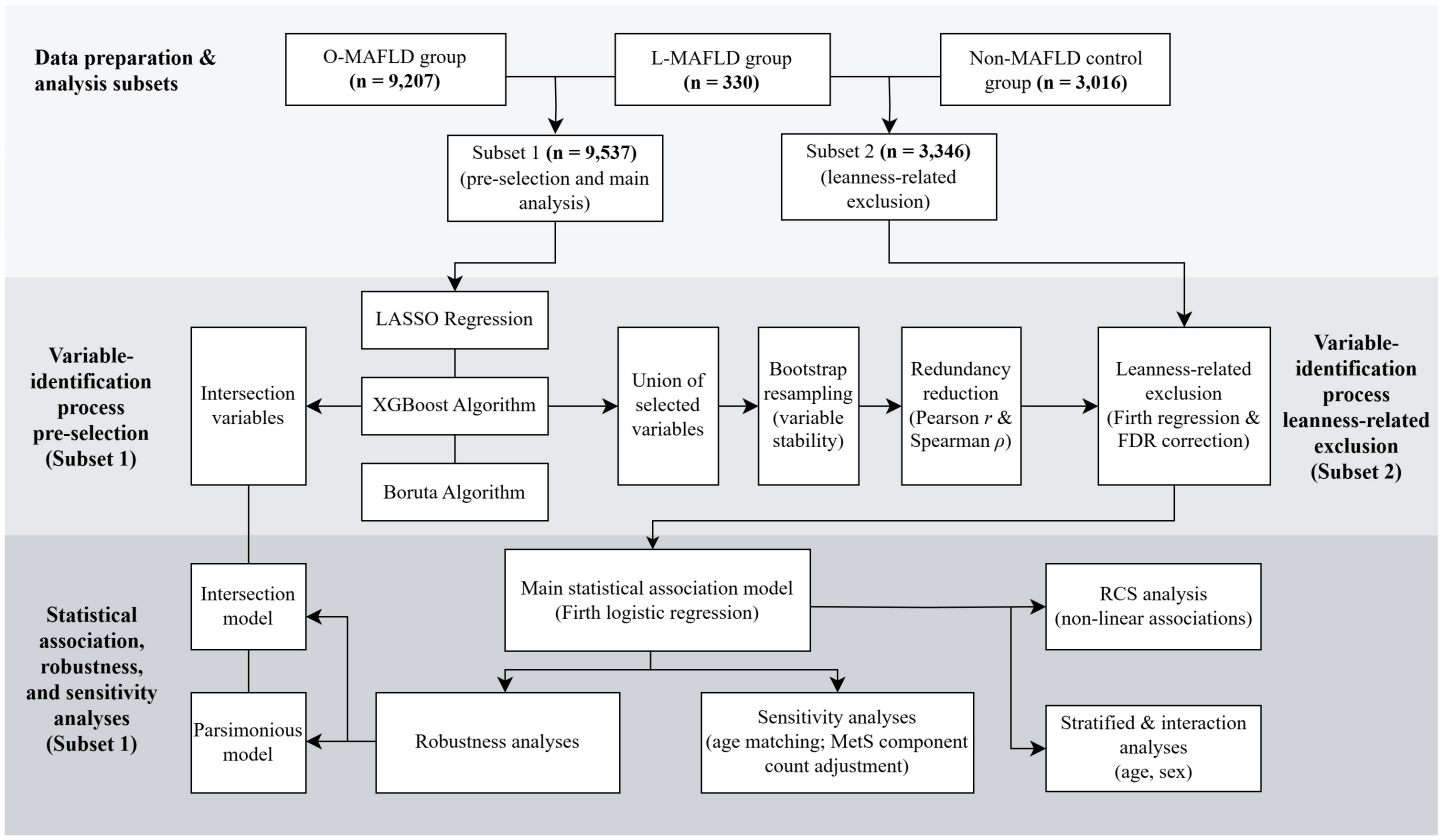
Study design and analytical framework. FDR, false discovery rate; L-MAFLD, lean metabolic dysfunction-associated fatty liver disease; LASSO, least absolute shrinkage and selection operator; MetS, metabolic syndrome; O-MAFLD, overweight/obese metabolic dysfunction-associated fatty liver disease; RCS, restricted cubic spline; XGBoost, extreme gradient boosting.

An integrated machine learning framework was used to identify biomarkers distinguishing L-MAFLD from O-MAFLD, followed by logistic regression to quantify statistical associations. All analyses were performed using R software (version 4.4.1), as illustrated in Figure 2.

The variable-identification process comprised pre-selection and leanness-related exclusion stages. During pre-selection, the L-MAFLD and O-MAFLD groups served as the primary dataset (Subset 1). Variables were initially selected using an ensemble of three methods: least absolute shrinkage and selection operator (LASSO), extreme gradient boosting (XGBoost), and the Boruta random forest wrapper algorithm. Bootstrap resampling was applied to evaluate variable stability. Pearson's *r* and Spearman's ρ were then calculated to reduce redundancy, yielding the final pre-selection variable set. To exclude variables that merely reflected lean body characteristics, the L-MAFLD and non-MAFLD control groups were combined into a secondary dataset (Subset 2). Each stable variable was independently evaluated in Subset 2, with multiple testing correction applied to identify the final variables. Machine learning was confined to the variable-identification process to ensure the subsequent regression models remained statistically interpretable.

In Subset 1, a multivariable logistic regression model was constructed as the main statistical association model. Robustness analyses were then performed using two alternative model specifications: a parsimonious model retaining only statistically significant variables; an intersection model including only variables selected by all three machine learning methods. Sensitivity analyses were further conducted using age-matched subsets and additional adjustment for the available MetS component count. Restricted cubic splines (RCS) were used to explore potential non-linear associations. Stratified and interaction analyses were conducted to assess potential effect modification by age and sex.

### 2.5 Machine learning techniques

A three-algorithm ensemble variable-selection framework was used to reduce single-model dependence. LASSO regression was used for sparse linear variable selection, XGBoost was used to capture nonlinear relationships and interactions, and the Boruta algorithm was used to identify variables with stable relevance (8). To ensure reproducibility, a fixed global random seed (20250606) was applied across all algorithms.

LASSO regression applies L1 regularization to penalize less important variables, shrinking their coefficients to zero to achieve model sparsity and variable selection (9). The LASSO model was constructed using the *glmnet* package (v4.1–8), which inherently standardizes continuous variables prior to fitting, utilizing binomial deviance as the evaluation metric. The optimal penalty parameter (λ) was determined via 10-fold cross-validation, selecting λ_1se_, the largest value within one standard error of the minimum, to define the non-zero coefficients.

XGBoost is an ensemble learning algorithm based on a gradient boosting framework that iteratively trains weak learners to fit residuals and minimize overall loss (10). XGBoost was implemented using the *xgboost* package (v1.7.0) with log loss as the evaluation metric. Hyperparameters were optimized via 5-fold cross-validation, with final settings established as: max_depth = 6, eta = 0.2, subsample = 0.8, colsample_bytree = 0.8, and nrounds = 300. Variable importance was assessed based on gain, and the top 30% of variables were retained.

Boruta is a random forest-based wrapper variable-selection method that compares the importance of original variables against created shadow features to identify variables with relevance exceeding that of random shadow features (11). Boruta analysis was conducted using the *Boruta* package (v7.0) with parameters set to ntree = 200 and maxRuns = 50. Variables classified as “Confirmed” were retained for subsequent analysis.

### 2.6 Statistical analysis

Before machine learning-based variable selection, missing data for the 26 candidate variables were addressed via multiple imputation by chained equations using the *mice* package (v3.17.0). Five imputed datasets were generated, and the results were pooled for subsequent analyses. For baseline comparisons, continuous variables were evaluated for normality using skewness (absolute value < 2) and kurtosis (absolute value < 7). Homogeneity of variance was assessed using Levene's test (α = 0.05). Normally distributed data with equal variances were compared using one-way ANOVA for three-group comparisons or independent Student's *t*-tests for two-group comparisons. For unequal variances, Welch's ANOVA and Welch's *t*-tests were applied. Non-normally distributed data were analyzed using the Kruskal-Wallis *H* test or Mann-Whitney *U* test. Categorical variables were compared using the χ^2^ test. Baseline characteristics are presented as counts and percentages, means and standard deviations, or medians and interquartile ranges, as appropriate.

To evaluate variable stability, 500 bootstrap resamples with replacement were performed on Subset 1. Variables selected by the machine learning algorithms in at least 80% of the resampling iterations were considered stable. To reduce redundancy, these stable variables were ranked in descending order of their selection frequency. Pearson's *r* and Spearman's ρ correlation coefficients were calculated pairwise; if the absolute correlation between a higher-ranked and a lower-ranked variable exceeded 0.7, the lower-ranked variable was excluded.

To address class imbalance and the limited sample size of the L-MAFLD group, Firth-penalized logistic regression was used to reduce small-sample bias and improve model stability. Robustness was evaluated using two alternative model specifications. A parsimonious model retaining only statistically significant variables was constructed to examine whether the direction and magnitude of the main associations remained stable after model simplification. An intersection model included only variables selected by all three machine learning methods. This model was compared with the main model using the likelihood ratio test to assess whether the additional variables in the main model improved model fit or were redundant.

To address potential residual confounding by age, an age-matched sensitivity analysis was performed between L-MAFLD participants and non-MAFLD controls. L-MAFLD cases were matched 1:1 to controls by nearest available age within a caliper of ± 3 years, without replacement. When multiple controls were eligible, the control with the smallest age difference was selected. Sex was used only as a tie-breaker. The parsimonious model was then refitted in the matched subset, with age and sex retained as covariates.

To assess whether biomarker associations were affected by the mandatory metabolic diagnostic criteria, an additional sensitivity analysis was conducted. The available MetS component count was added to the previously established parsimonious model comparing L-MAFLD with O-MAFLD. This conservative available-component count ranged from 0 to 4 and was built from four observed components: elevated blood pressure (SBP ≥ 130 mmHg or DBP ≥ 85 mmHg), elevated triglycerides (TG ≥ 1.7 mmol/L), reduced HDL-C (HDL-C < 1.0 mmol/L in men or < 1.3 mmol/L in women), and elevated fasting glucose (FBG ≥ 5.6 mmol/L).

RCS models fitted with four knots were used to explore potential non-linear associations for variables with significant main effects. Stratified and interaction analyses were conducted by sex and by age group, with age divided at the median. To control the false discovery rate (FDR), *P* values from univariate, stratified, and interaction analyses were adjusted using the Benjamini-Hochberg method. Statistical significance was defined as a two-sided *P* < 0.05.

## 3 Results

### 3.1 Baseline characteristics

Compared with the O-MAFLD group, the L-MAFLD group had a higher proportion of females (*P* < 0.001) and an older mean age (*P* < 0.001). The L-MAFLD group also showed higher levels of FBG, HbA1c, HDL-C, and pulse rate (all *P* < 0.001). In contrast, it had lower levels of the ALT/AST ratio, UA, Scr, ALP, CK, LDH, GGT, DBil, WBC, RBC, Hb, and FT3 (all *P* < 0.001), as well as DBP, IBil, CysC, TBil, PAB, and FT4 (all *P* < 0.05).

Compared with the non-MAFLD control group, the L-MAFLD group had a higher proportion of males (*P* < 0.001) and an older mean age (*P* < 0.001). It also showed higher levels of SBP, DBP, TC, TG, LDL-C, FBG, HbA1c, the ALT/AST ratio, CysC, UA, BUN, chloride, Scr, calcium, sodium, α-HBDH, ALP, PAB, CK, LDH, GGT, DBil, RBC, WBC, Hb, and FT3 (all *P* < 0.05). HDL-C, phosphorus, FT4, and TSH were lower in the L-MAFLD group (all *P* < 0.05). Detailed baseline characteristics are summarized in Table 1.

**Table 1:** Baseline characteristics of the non-MAFLD control, O-MAFLD, and L-MAFLD groups.

Variable	non–MAFLD Control group (*n* = 3,016)	O–MAFLD group (*n* = 9,207)	L–MAFLD group (*n* = 330)	Test statistic (χ^2^/*H*/*F*)	*P* value
Sex [*n* (%)]				2,974.00^a^	< 0.001
Male	615 (20.39)	6,985 (75.87)	182 (55.15)^cd^		
Female	2,401 (79.61)	2,222 (24.13)	148 (44.85)^cd^		
Age (years)	37 ± 12	47 ± 13	53 ± 13^cd^	826.68	< 0.001
Pulse (beats/min)	83 ± 12	81 ± 12	83 ± 13^c^	22.48	< 0.001
Systolic blood pressure (mmHg)	111 ± 10	134 ± 17	134 ± 18^d^	4,056.47	< 0.001
Diastolic blood pressure (mmHg)	67 ± 8	82 ± 11	80 ± 11^cd^	3,105.1	< 0.001
Total cholesterol (mmol/L)	4.51 ± 0.79	4.88 ± 0.95	4.97 ± 0.99^d^	240.61	< 0.001
Lipoprotein(a) (mg/L)	163.1 (104.5, 311.3)	147.7 (96.3, 260.4)	153.8 (101.7, 248.2)	44.17^b^	< 0.001
Triglycerides (mmol/L)	0.80 (0.63, 1.02)	1.84 (1.33, 2.63)	1.87 (1.43, 2.61)^d^	4,915.17^b^	< 0.001
HDL–C (mmol/L)	1.64 ± 0.29	1.13 ± 0.26	1.21 ± 0.29^cd^	3,492.51	< 0.001
LDL–C (mmol/L)	2.68 ± 0.70	3.09 ± 0.84	3.06 ± 0.85^d^	355.11	< 0.001
Fasting blood glucose (mmol/L)	4.72 (4.46, 4.98)	5.21 (4.84, 5.77)	5.36 (4.95, 6.23)^cd^	2,146.28^b^	< 0.001
HbA1c (%)	5.3 (5.1, 5.5)	5.7 (5.4, 6.1)	5.8 (5.5, 6.3)^cd^	2,734.17^b^	< 0.001
Globulin (g/L)	25.4 ± 3.3	25.8 ± 3.3	25.7 ± 3.3	10.49	< 0.001
Indirect bilirubin (μmol/L)	6.4 (4.5, 8.7)	7.2 (5.3, 9.8)	6.7 (4.9, 9.1)^c^	178.43^b^	< 0.001
ALT/AST ratio	0.73 (0.61, 0.90)	1.20 (0.96, 1.52)	1.03 (0.84, 1.23) ^cd^	3,516.84^b^	< 0.001
Potassium (mmol/L)	4.14 ± 0.28	4.18 ± 0.30	4.17 ± 0.33	20.67	< 0.001
Cystatin C (mg/L)	0.73 (0.65, 0.81)	0.85 (0.76, 0.95)	0.82 (0.74, 0.92)^cd^	1,592.86^b^	< 0.001
Total bilirubin (μmol/L)	10.4 (7.9, 13.8)	11.4 (8.7, 14.9)	10.6 (7.9, 13.8)^c^	118.56^b^	< 0.001
Uric acid (μmol/L)	261.9 ± 63.6	364.3 ± 89.3	323.2 ± 80.7^cd^	2,373.83	< 0.001
Blood urea nitrogen (mg/dL)	13.50 ± 3.45	15.01 ± 3.70	14.90 ± 3.81^d^	213.53	< 0.001
Chloride (mmol/L)	103.63 ± 2.01	102.85 ± 2.44	103.10 ± 2.57^d^	154.09	< 0.001
Serum creatinine (μmol/L)	61.1 ± 11.5	72.8 ± 14.5	65.9 ± 15.1^cd^	1,035.92	< 0.001
Calcium (mmol/L)	2.34 ± 0.08	2.36 ± 0.09	2.37 ± 0.09^d^	105.49	< 0.001
Albumin (g/L)	47.1 ± 2.5	47.5 ± 2.6	47.3 ± 2.5	34.16	< 0.001
Sodium (mmol/L)	140.27 ± 1.79	140.69 ± 1.90	140.85 ± 2.05^d^	61.77	< 0.001
HBDH (U/L)	122.0 (111.0, 135.0)	131.0 (119.0, 146.0)	130.0 (118.0, 144.8)^d^	504.49^b^	< 0.001
Phosphorus (mmol/L)	1.18 ± 0.15	1.11 ± 0.16	1.13 ± 0.16^d^	189.54	< 0.001
Alkaline phosphatase (U/L)	60.0 (50.0, 71.0)	76.0 (64.0, 90.0)	82.0 (62.3, 97.8)^cd^	1,567.66^b^	< 0.001
Prealbumin (mg/L)	256.5 ± 52.9	323.9 ± 71.2	314.1 ± 70.4^cd^	1,546.91	< 0.001
Creatine kinase (U/L)	70.0 (55.0, 93.0)	93.0 (72.0, 126.0)	75.5 (60.0, 100.0)^cd^	932.83^b^	< 0.001
Lactate dehydrogenase (U/L)	159.0 (144.0, 176.0)	176.0 (159.0, 195.0)	169.0 (156.0, 187.8)^cd^	890.47^b^	< 0.001
GGT (U/L)	12.0 (10.0, 16.0)	32.0 (22.0, 51.0)	25.0 (18.0, 40.0)^cd^	4,286.53^b^	< 0.001
Direct bilirubin (μmol/L)	4.1 (3.2, 5.1)	4.2 (3.3, 5.3)	3.9 (3.1, 4.8)^cd^	25.21^b^	< 0.001
WBC count ( × 10^9^/L)	5.56 ± 1.40	6.51 ± 1.62	6.12 ± 1.39^cd^	480.03	< 0.001
RBC count ( × 10^12^/L)	4.49 ± 0.41	5.03 ± 0.46	4.82 ± 0.48^cd^	1,856.78	< 0.001
Hemoglobin (g/L)	134.3 ± 14.3	152.5 ± 14.9	145.4 ± 15.0^cd^	1,800.13	< 0.001
FT3 (pmol/L)	4.87 (4.47, 5.29)	5.36 (4.93, 5.83)	5.04 (4.68, 5.50)^cd^	1,237.44^b^	< 0.001
FT4 (pmol/L)	16.70 (15.30, 18.10)	16.80 (15.30, 18.60)	16.60 (14.90, 18.20)^cd^	17.34^b^	< 0.001
TSH (mIU/L)	2.16 (1.48, 3.10)	2.06 (1.48, 2.93)	2.03 (1.31, 3.10)^d^	14.93^b^	0.001

^a^ Chi–squared test; ^b^ Kruskal–Wallis test; unmarked statistics derived from ANOVA. ^c^ *P* < 0.05 vs O–MAFLD group; ^d^ *P* < 0.05 vs non–MAFLD control group.

ALT, alanine aminotransferase; AST, aspartate aminotransferase; FT3, free triiodothyronine; FT4, free thyroxine; GGT, γ-glutamyl transferase; HbA1c, hemoglobin A1c; HBDH, α-hydroxybutyrate dehydrogenase; HDL–C, high–density lipoprotein cholesterol; LDL–C, low–density lipoprotein cholesterol; L–MAFLD, lean metabolic dysfunction–associated fatty liver disease; O–MAFLD, overweight/obese metabolic dysfunction–associated fatty liver disease; RBC, red blood cell; TSH, thyroid–stimulating hormone; WBC, white blood cell.

### 3.2 Machine learning-based variable selection

**Figure 3:**
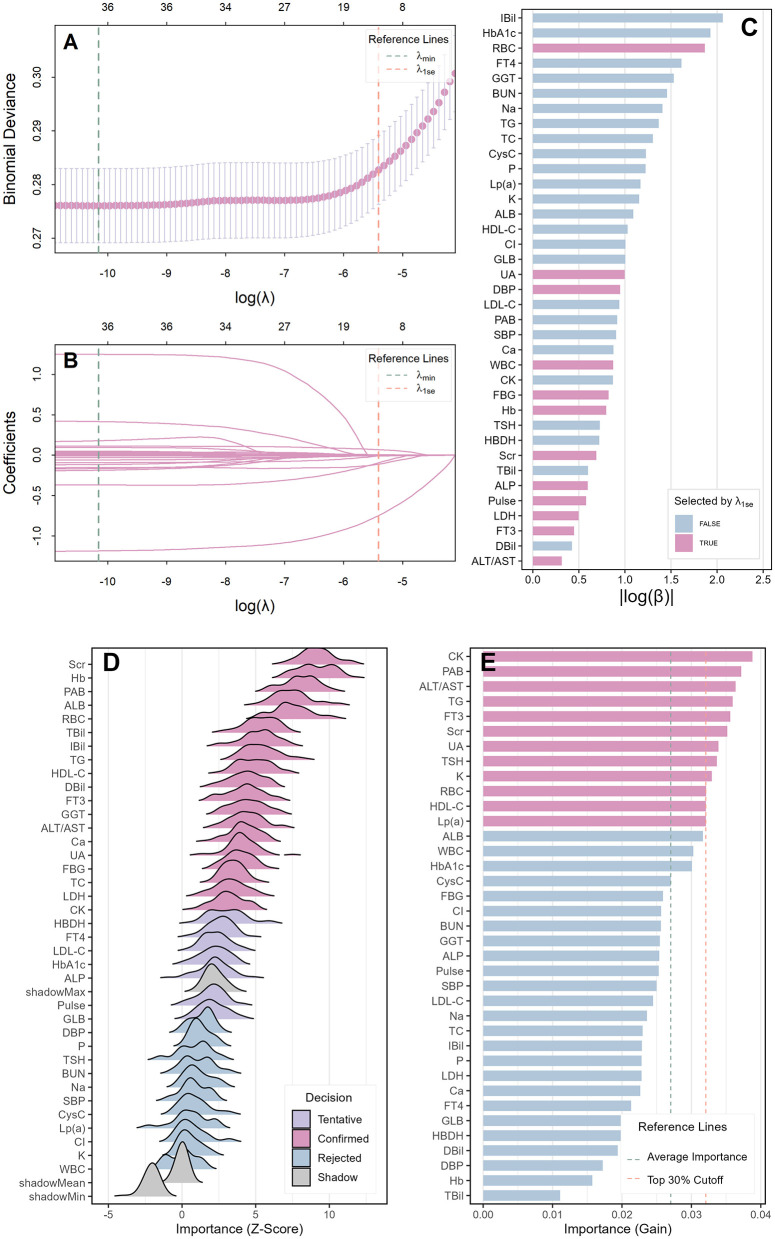
Machine learning-based variable identification. **(A)** LASSO regression cross-validation error curve. Red dots indicate mean error; purple shading represents the error range; the orange dashed line marks the optimal regularization threshold (λ_1se_). **(B)** LASSO coefficient profiles. The solid lines visualize how variable coefficients shrink toward zero as regularization (log(λ)) increases. **(C)** Variables selected by LASSO based on absolute logistic regression coefficients (|logβ|). Red bars indicate selected variables. **(D)** Variable importance plot generated by the Boruta algorithm. Red, purple, and blue indicate confirmed, tentative, and rejected variables, respectively. **(E)** XGBoost variable importance ranking, with the orange dashed line marking the top 30% importance threshold. ALB, albumin; ALP, alkaline phosphatase; ALT/AST, alanine aminotransferase to aspartate aminotransferase ratio; BUN, blood urea nitrogen; Ca, calcium; CK, creatine kinase; Cl, chloride; CysC, cystatin C; DBil, direct bilirubin; DBP, diastolic blood pressure; FBG, fasting blood glucose; FT3, free triiodothyronine; FT4, free thyroxine; GGT, γ-glutamyl transferase; GLB, globulin; Hb, hemoglobin; HbA1c, hemoglobin A1c; HBDH, α-hydroxybutyrate dehydrogenase; HDL-C, high-density lipoprotein cholesterol; IBil, indirect bilirubin; K, potassium; LDH, lactate dehydrogenase; LDL-C, low-density lipoprotein cholesterol; Lp(a), lipoprotein(a); Na, sodium; P, phosphorus; PAB, prealbumin; RBC, red blood cell count; SBP, systolic blood pressure; Scr, serum creatinine; TBil, total bilirubin; TC, total cholesterol; TG, triglycerides; TSH, thyroid-stimulating hormone; UA, uric acid; WBC, white blood cell count.

The optimal tuning parameter (λ_1se_) for the LASSO model was identified via 10-fold cross-validation (Figure 3A), with coefficient profiles visualized against the log(λ) sequence (Figure 3B). At the optimal λ_1se_ threshold, LASSO regression identified the following variables: pulse, DBP, FBG, the ALT/AST ratio, UA, Scr, ALP, LDH, WBC, RBC, Hb, and FT3 (Figure 3C). The XGBoost algorithm retained the top 30% of variables based on importance scores: CK, PAB, the ALT/AST ratio, TG, FT3, Scr, UA, TSH, potassium, RBC, HDL-C, and Lp(a) (Figure 3E). The Boruta algorithm identified the following relevant variables: TC, TG, HDL-C, FBG, IBil, the ALT/AST ratio, TBil, UA, Scr, calcium, ALB, PAB, CK, LDH, GGT, DBil, RBC, Hb, and FT3 (Figure 3D). The ALT/AST ratio, UA, Scr, RBC, and FT3 were consistently selected across all three algorithms.

### 3.3 Variable stability, redundancy reduction, and leanness-related exclusion

An initial set of 26 candidate variables was identified using the machine learning framework. Stability was assessed via bootstrap resampling, with all variables achieving a selection frequency of ≥ 80%. After ranking the variables by selection frequency, redundancy was evaluated using Pearson's *r* and Spearman's ρ correlation coefficients. Redundant variables highly correlated with higher-ranked variables (e.g., TBil, DBil, and Hb) were excluded. A total of 23 stable and non-redundant variables were retained. In Subset 2, univariable Firth-penalized logistic regression was performed for each stable and non-redundant variable. After FDR adjustment, 19 variables were retained for the final multivariable main model (Table 2).

**Table 2:** Variable retention during stability assessment, redundancy reduction, and leanness–related exclusion.

Variable	Identified by machine learning (*n* = 26)	Stable after bootstrap assessment (*n* = 26)	Retained after redundancy reduction (*n* = 23)	Retained after exclusion in Subset 2 (*n* = 19)
Pulse	✓	✓	✓	✓
Diastolic blood pressure (DBP)	✓	✓	✓	✓
Fasting blood glucose (FBG)	✓	✓	✓	✓
ALT/AST ratio	✓	✓	✓	✓
Uric acid (UA)	✓	✓	✓	✓
Serum creatinine (Scr)	✓	✓	✓	✓
Alkaline phosphatase (ALP)	✓	✓	✓	✓
Creatine kinase (CK)	✓	✓	✓	
Prealbumin (PAB)	✓	✓	✓	✓
Albumin (ALB)	✓	✓	✓	✓
γ-glutamyl transferase (GGT)	✓	✓	✓	✓
Lactate dehydrogenase (LDH)	✓	✓	✓	
Total bilirubin (TBil)	✓	✓		
Direct bilirubin (DBil)	✓	✓		
Indirect bilirubin (IBil)	✓	✓	✓	✓
Total cholesterol (TC)	✓	✓	✓	✓
Triglycerides (TG)	✓	✓	✓	✓
High–density lipoprotein cholesterol (HDL–C)	✓	✓	✓	✓
Lipoprotein(a) [Lp(a)]	✓	✓	✓	✓
Calcium	✓	✓	✓	✓
Potassium	✓	✓	✓	
Hemoglobin (Hb)	✓	✓		
Red blood cell count (RBC)	✓	✓	✓	✓
White blood cell count (WBC)	✓	✓	✓	✓
Free triiodothyronine (FT3)	✓	✓	✓	✓
Thyroid–stimulating hormone (TSH)	✓	✓	✓	

✓ indicates that the variable was selected or retained at the corresponding step.

ALT, alanine aminotransferase; AST, aspartate aminotransferase.

### 3.4 Multivariable regression, robustness, and sensitivity analyses

In the main multivariable model, higher pulse rate and higher levels of FBG, ALP, and PAB were associated with higher odds of L-MAFLD (all *P* < 0.05). In contrast, higher ALT/AST ratio, Scr, FT3, DBP, WBC, and UA were associated with lower odds of L-MAFLD (all *P* < 0.05). Detailed odds ratios (ORs) and 95% confidence intervals (CIs) are summarized in Table 3.

**Table 3:** Multivariable Firth–penalized logistic regression analysis of factors associated with L–MAFLD.

Variable	Main model		Parsimonious model		Intersection model	
	OR (95% CI)	*P* value	OR (95% CI)	*P* value	OR (95% CI)	*P* value
Age	1.014 (1.003–1.025)	0.010	1.014 (1.004–1.025)	0.007	1.013 (1.003–1.023)	0.008
Sex	1.269 (0.871–1.847)	0.214	1.077 (0.765–1.517)	0.671	1.137 (0.804–1.606)	0.469
ALT/AST ratio	0.345 (0.238–0.497)	< 0.001	0.338 (0.234–0.483)	< 0.001	0.394 (0.275–0.561)	< 0.001
Free triiodothyronine	0.708 (0.582–0.860)	< 0.001	0.711 (0.587–0.860)	< 0.001	0.734 (0.606–0.887)	0.001
Serum creatinine	0.981 (0.969–0.992)	0.001	0.980 (0.969–0.991)	< 0.001	0.978 (0.966–0.989)	< 0.001
Uric acid	0.998 (0.997–1.000)	0.040	0.998 (0.997–1.000)	0.068	0.998 (0.997–1.000)	0.052
Pulse rate	1.021 (1.011–1.031)	< 0.001	1.023 (1.013–1.032)	< 0.001	—	—
Alkaline phosphatase	1.010 (1.005–1.015)	< 0.001	1.010 (1.005–1.015)	< 0.001	—	—
Diastolic blood pressure	0.980 (0.969–0.990)	< 0.001	0.979 (0.969–0.990)	< 0.001	—	—
White blood cell count	0.897 (0.828–0.970)	0.006	0.898 (0.830–0.969)	0.006	—	—
Fasting blood glucose	1.081 (1.017–1.145)	0.014	1.089 (1.026–1.151)	0.006	—	—
Prealbumin	1.002 (1.000–1.004)	0.029	1.003 (1.001–1.005)	0.002	—	—
Red blood cell count	0.777 (0.566–1.069)	0.121	—	—	0.858 (0.633–1.164)	0.324
Calcium	3.320 (0.815–13.024)	0.094	—	—	—	—
Triglycerides	1.049 (0.969–1.128)	0.231	—	—	—	—
Indirect bilirubin	0.982 (0.950–1.014)	0.274	—	—	—	—
Lipoprotein(a)	1.000 (0.999–1.000)	0.330	—	—	—	—
Albumin	1.024 (0.971–1.081)	0.380	—	—	—	—
High–density lipoprotein cholesterol	1.177 (0.705–1.932)	0.528	—	—	—	—
Total cholesterol	0.968 (0.846–1.107)	0.638	—	—	—	—
γ-glutamyl transferase	1.000 (0.997–1.002)	0.896	—	—	—	—

All models were adjusted for age and sex. The main model included 19 candidate variables. The parsimonious model retained variables that were statistically significant in the main model. The intersection model included variables identified by all three machine learning methods. “—” indicates variable was not included in the model. ALT, alanine aminotransferase; AST, aspartate aminotransferase; CI, confidence interval; OR, odds ratio.

In the parsimonious model, the effect directions remained consistent with the main model. The 95% CIs also overlapped for most variables. UA became marginally significant (*P* = 0.068), while the other variables remained statistically significant. The intersection model differed significantly from the main model (likelihood ratio χ^2^ = 165.603, *P* < 0.001). This suggested that the additional variables retained in the main model contributed to model fit rather than being redundant.

In the age-matched sensitivity analysis, 318 of 330 L-MAFLD cases were matched to 318 non-MAFLD controls. Age balance improved substantially after matching. In the matched subset, several biomarkers retained in the parsimonious model remained statistically significant, including Scr, FBG, ALT/AST ratio, UA, ALP, WBC, and PAB. FT3 was no longer statistically significant (Supplementary Table S1). In an additional sensitivity model, the available MetS component count was added to the previously established parsimonious multivariable model comparing L-MAFLD with O-MAFLD. Scr, FT3, PAB, ALT/AST ratio, ALP, WBC, UA, DBP, and pulse rate remained statistically associated with L-MAFLD. The association with FBG was attenuated and was no longer statistically significant (Supplementary Table S2).

### 3.5 Restricted cubic spline analysis

**Figure 4:**
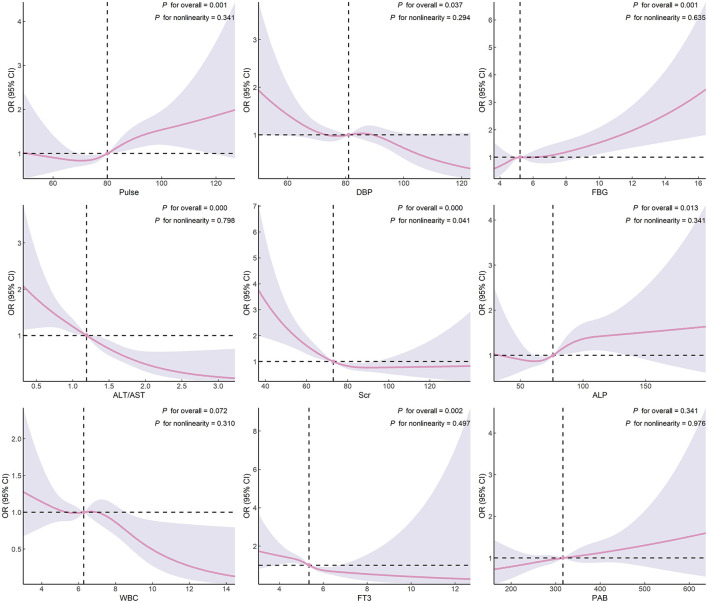
Restricted cubic spline curves for identified variables. ALP, alkaline phosphatase; ALT/AST, alanine aminotransferase to aspartate aminotransferase ratio; CI, confidence interval; DBP, diastolic blood pressure; FBG, fasting blood glucose; FT3, free triiodothyronine; OR, odds ratio; PAB, prealbumin; Pulse, pulse rate; Scr, serum creatinine; WBC, white blood cell count.

RCS models fitted with four knots were used to explore potential non-linear associations between the identified significant variables and the odds of L-MAFLD (Figure 4). WBC and PAB showed no statistically significant overall associations with L-MAFLD (*P* for overall association = 0.072 and 0.341, respectively). The ALT/AST ratio, FT3, ALP, FBG, pulse rate, and DBP showed no evidence of non-linearity (all *P* for non-linearity > 0.05). In contrast, Scr showed a statistically significant non-linear association with L-MAFLD (*P* for non-linearity = 0.041). The curve showed an L-shaped pattern, with a rapid initial decrease in odds followed by a plateau. The inflection point was approximately 60 μmol/L (OR = 1.586, 95% CI 1.282–1.963).

### 3.6 Stratified and interaction analyses

**Figure 5:**
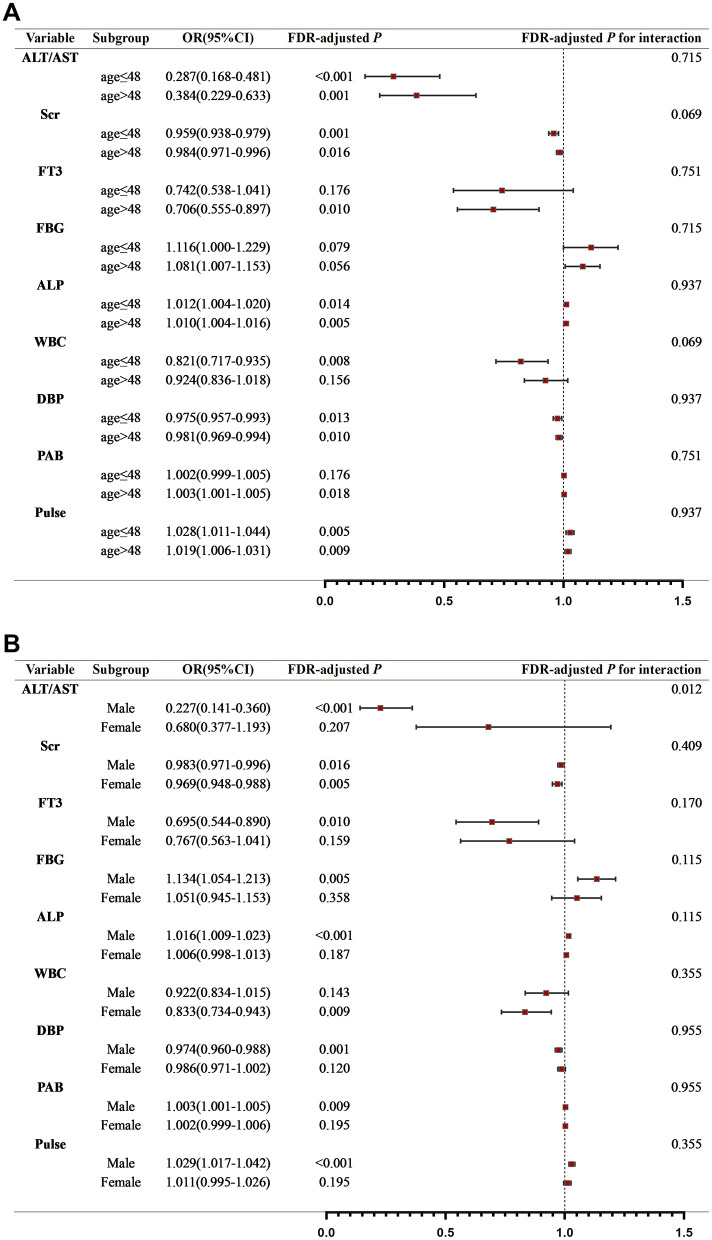
Forest plots of stratified and interaction analyses. **(A)** Analysis stratified by median age. **(B)** Analysis stratified by sex. ALP, alkaline phosphatase; ALT/AST, alanine aminotransferase to aspartate aminotransferase ratio; CI, confidence interval; DBP, diastolic blood pressure; FBG, fasting blood glucose; FT3, free triiodothyronine; OR, odds ratio; PAB, prealbumin; Pulse, pulse rate; Scr, serum creatinine; WBC, white blood cell count.

In the analysis stratified by the median age of 48 years (Figure 5A), FT3 and PAB were significantly associated with L-MAFLD only in participants aged > 48 years, whereas WBC was significant only in those aged ≤ 48 years. The associations for the ALT/AST ratio, Scr, ALP, DBP, and pulse rate remained statistically significant across both age strata (all *P* < 0.05). Interaction analyses showed no significant effect modification by any of these variables (all *P* for interaction > 0.05).

In the sex-stratified analysis (Figure 5B), WBC was significantly associated with L-MAFLD only in females, whereas the ALT/AST ratio, FT3, FBG, ALP, DBP, PAB, and pulse rate were significant only in males. Scr remained significantly associated with L-MAFLD in both sexes (all *P* < 0.05). A significant sex-specific interaction was observed only for the ALT/AST ratio (*P* for interaction = 0.012). No other variables showed significant effect modification by sex (all other *P* for interaction > 0.05).

## 4 Discussion

The liver is central to carbohydrate and lipid metabolism. IR and metabolic dysfunction can promote fatty acid synthesis and storage, impair lipid export, and contribute to hepatic steatosis. The nomenclature of MAFLD emphasizes metabolic dysfunction, reflecting the concept that hepatic steatosis is part of systemic metabolic disorder rather than isolated fat deposition (12, 13). Previous epidemiological studies have found that L-MAFLD accounts for a substantial proportion of MAFLD cases in Asian populations (14). Long-term cohort studies have shown that individuals with L-MAFLD may experience faster liver fibrosis progression and higher risks of cardiovascular events and all-cause mortality than those with O-MAFLD (5, 15). These findings suggest that L-MAFLD may represent a metabolically unfavorable phenotype despite the absence of overt obesity. Many previous studies have focused on comparisons between MAFLD and non-MAFLD populations or on obesity-related MAFLD. In this study, we focused on L-MAFLD and used an integrated machine learning framework to identify routine biomarker features. Our results characterize a biomarker profile for L-MAFLD across hepatobiliary, renal, cardiovascular, and endocrine systems.

A relatively low ALT/AST ratio was associated with L-MAFLD, with a stronger inverse association in males. A decreased ALT/AST ratio may indirectly reflect increased AST release into the blood. AST is a key enzyme in the malate-aspartate shuttle. This pattern may suggest a potential link between L-MAFLD and mitochondrial dysfunction or altered β-oxidation (16). Previous basic research suggests that sex hormone pathways may modulate hepatic mitochondrial biogenesis and lipid antioxidant capacity in different ways (17–20). This may partly explain the sex-specific heterogeneity observed in this study.

Our analysis showed an inverse association between Scr and L-MAFLD. Although Scr is primarily a renal function marker, it is also used in population-based studies as an indirect surrogate for skeletal muscle mass (21). The L-shaped association, in which the odds of L-MAFLD increased as Scr decreased to approximately 60 μmol/L, is compatible with a possible link between L-MAFLD and lower skeletal muscle reserve. Recent clinical evidence has highlighted crosstalk between muscle and liver. Lower muscle mass may aggravate IR and metabolic complications (22, 23). However, Scr is influenced by renal function, dietary protein and meat intake (24), and other nutritional factors. Direct body composition, dietary protein intake, vegetarian status, and supplement use were unavailable. This inflection point may also reflect early glomerular hyperfiltration in metabolic disorders (25). The muscle reserve interpretation therefore remains preliminary and should be tested with direct body composition or muscle function measures.

Lower FT3 was associated with L-MAFLD in the main model, with associations observed mainly in older and male subgroups. Thyroid hormones help maintain basal metabolism and regulate fatty acid oxidation (26, 27). In obesity-associated O-MAFLD, leptin-related activation of the hypothalamic-pituitary-thyroid axis may slightly increase triiodothyronine levels. The absence of an obesity-related compensatory thyroid response may partly explain the lower FT3 observed in L-MAFLD (28). FT3 was retained in the main multivariable model and in the comparison additionally adjusted for available MetS component count, but not after age matching. This suggests sensitivity to age structure. Future studies should evaluate the association between FT3 and L-MAFLD in age-balanced cohorts and determine whether it relates to metabolic progression or fibrosis outcomes.

Differences in basic physiological indicators, such as decreased DBP and lower WBC counts, were also associated with L-MAFLD. Autonomic nervous system imbalance and differences in systemic inflammation may partly explain these findings (29, 30). These parameters are also susceptible to clinical fluctuation. Unmeasured lifestyle factors, including dietary patterns, physical activity, and the use of antihypertensive or anti-inflammatory medications, may have contributed to these differences. The concurrent elevation of ALP and FBG also points to a systemic metabolic context. ALP may reflect hepatobiliary or bone metabolic involvement, whereas FBG is closely tied to glucose metabolic burden (30–32). FBG was attenuated after adjustment for the available MetS component count. Its association with L-MAFLD may therefore reflect overall metabolic burden rather than a lean-specific process. This interpretation is consistent with evidence that lean fatty liver disease may precede MetS development (33).

This study provides useful information on the clinical phenotyping of L-MAFLD, but its cross-sectional design prevents causal inference. Hepatic ultrasonography is practical for large-scale epidemiological studies, but its sensitivity for mild steatosis is lower than that of liver biopsy or magnetic resonance imaging (34). A key diagnostic difference exists in the current MAFLD criteria. Non-obese individuals require type 2 diabetes or at least two metabolic abnormalities for diagnosis, whereas overweight or obese individuals are diagnosed based on BMI plus hepatic steatosis. Therefore, some identified biomarkers may reflect broader metabolic burden rather than processes specific to lean status. Although heavy alcohol intake was excluded, quantitative alcohol consumption among light-to-moderate drinkers was unavailable and could not be further adjusted for, leaving potential residual confounding (35). Age matching greatly improved age balance, but residual confounding by age cannot be fully excluded. The MetS sensitivity analysis used a score based on available components rather than a complete metabolic burden measure. The interpretation is also limited by the absence of direct measurements for skeletal muscle mass, dietary protein intake, vegetarian status, supplement use, IR, and medication history (24, 36). Future longitudinal multicenter studies should include direct body composition assessment, dietary data, and comprehensive metabolic risk profiles. Independent external validation is needed to assess whether this biomarker profile is reproducible in other populations.

## 5 Conclusion

This study characterizes a distinct biomarker profile of L-MAFLD. Several routine biomarkers across hepatobiliary, renal, cardiovascular, metabolic, and inflammatory domains were associated with L-MAFLD. The ALT/AST ratio showed heterogeneity by sex, and Scr showed an L-shaped association. These findings remain association-based and should not be interpreted as evidence for immediate clinical use. Future longitudinal multicenter studies with more complete metabolic profiling and external validation are needed.

## Data Availability

The datasets presented in this article are not readily available due to hospital regulations regarding patient privacy. Requests to access the datasets should be directed to the corresponding author.
