# Supplementary material for: Clinical and biochemical phenotypes of Lean MAFLD: machine learning-based biomarker identification and statistical association analysis in a cross-sectional study
Source: Front Public Health. 2026 Jul 31;14:1835144. doi: 10.3389/fpubh.2026.1835144 (PMC13473254; doi:10.3389/fpubh.2026.1835144)
Supplement: Supplementary file 1 [file Table_1.docx]

**Supplementary Table S1. Age-matched sensitivity analysis comparing L-MAFLD with non-MAFLD controls.**

**Panel A. Age balance before and after 1:1 age matching.**

| **Matching status** | **Group** | ***n*** | **Age, mean ± SD** | **Age, median (range)** | **Male, *n* (%)** | **Age SMD** |
| --- | --- | --- | --- | --- | --- | --- |
| Before matching | Non-MAFLD controls | 3016 | 37.0 ± 11.5 | 34 (18-79) | 615 (20.4) | 1.287 |
| Before matching | L-MAFLD | 330 | 52.7 ± 12.9 | 54 (23-86) | 182 (55.2) | 1.287 |
| After matching | Non-MAFLD controls | 318 | 51.7 ± 11.8 | 53 (23-79) | 178 (56.0) | 0.003 |
| After matching | L-MAFLD | 318 | 51.6 ± 11.8 | 53 (23-79) | 173 (54.4) | 0.003 |

**Panel B. Biomarkers retained in the parsimonious model in the age-matched subset.**

| **Variable** | **OR** | **95% CI** | ***P* value** |
| --- | --- | --- | --- |
| Pulse rate | 0.985 | 0.964-1.006 | 0.161 |
| DBP | 1.149 | 1.114-1.185 | <0.001 |
| FBG | 3.419 | 2.195-5.328 | <0.001 |
| ALT/AST ratio | 4.972 | 2.178-11.350 | <0.001 |
| UA | 1.013 | 1.009-1.017 | <0.001 |
| Scr | 0.970 | 0.945-0.995 | 0.021 |
| ALP | 1.019 | 1.007-1.031 | 0.001 |
| WBC | 1.486 | 1.240-1.781 | <0.001 |
| FT3 | 0.978 | 0.800-1.195 | 0.824 |
| PAB | 1.007 | 1.002-1.011 | 0.004 |

**Notes:** L-MAFLD cases were matched 1:1 to non-MAFLD controls by nearest available age within a ±3-year caliper, without replacement; sex was used only as a tie-breaker when age differences were equal. The age-matched logistic regression model included the biomarkers retained in the parsimonious model shown in Panel B, with further adjustment for age and sex. Age SMD was calculated as the absolute difference in group means divided by the pooled standard deviation. **Abbreviations:** ALP, alkaline phosphatase; ALT, alanine aminotransferase; AST, aspartate aminotransferase; CI, confidence interval; DBP, diastolic blood pressure; FBG, fasting blood glucose; FT3, free triiodothyronine; L-MAFLD, lean metabolic dysfunction-associated fatty liver disease; O-MAFLD, overweight/obese metabolic dysfunction-associated fatty liver disease; OR, odds ratio; PAB, prealbumin; Scr, serum creatinine; SD, standard deviation; SMD, standardized mean difference; UA, uric acid; WBC, white blood cell count.
