# Supplementary material for: Clinical and biochemical phenotypes of Lean MAFLD: machine learning-based biomarker identification and statistical association analysis in a cross-sectional study
Source: Front Public Health. 2026 Jul 31;14:1835144. doi: 10.3389/fpubh.2026.1835144 (PMC13473254; doi:10.3389/fpubh.2026.1835144)
Supplement: Supplementary file 2 [file Table_2.docx]

**Supplementary Table S2. Sensitivity analysis of the parsimonious multivariable model comparing L-MAFLD with O-MAFLD after additional adjustment for available MetS component count.**

| **Variable** | **OR** | **95% CI** | ***P* value** |
| --- | --- | --- | --- |
| Scr | 0.980 | 0.969-0.991 | <0.001 |
| FT3 | 0.705 | 0.583-0.853 | <0.001 |
| PAB | 1.003 | 1.001-1.004 | 0.005 |
| ALT/AST ratio | 0.315 | 0.218-0.454 | <0.001 |
| ALP | 1.009 | 1.004-1.014 | <0.001 |
| WBC | 0.886 | 0.819-0.958 | 0.003 |
| UA | 0.998 | 0.996-1.000 | 0.026 |
| DBP | 0.973 | 0.963-0.984 | <0.001 |
| Pulse rate | 1.022 | 1.012-1.032 | <0.001 |
| FBG | 1.048 | 0.984-1.116 | 0.147 |

**Notes:** The sensitivity analysis was based on the previously established parsimonious multivariable model comparing L-MAFLD with O-MAFLD, with additional adjustment for the available MetS component count.

**Abbreviations:** ALP, alkaline phosphatase; ALT, alanine aminotransferase; AST, aspartate aminotransferase; CI, confidence interval; DBP, diastolic blood pressure; FBG, fasting blood glucose; FT3, free triiodothyronine; L-MAFLD, lean metabolic dysfunction-associated fatty liver disease; MetS, metabolic syndrome; O-MAFLD, overweight/obese metabolic dysfunction-associated fatty liver disease; OR, odds ratio; PAB, prealbumin; Scr, serum creatinine; UA, uric acid; WBC, white blood cell count.
